# Serp-1 Promotes Corneal Wound Healing by Facilitating Re-epithelialization and Inhibiting Fibrosis and Angiogenesis

**DOI:** 10.3389/fcvm.2021.649124

**Published:** 2021-06-04

**Authors:** Brent Ju, Owen Guo, Dathe Z. Benissan-Messan, McKinley H. Shawver, Peng Chen, Bingchuan Geng, Siqi Wei, Jordan R. Yaron, Alexandra R. Lucas, Hua Zhu

**Affiliations:** ^1^Division of Cardiac Surgery, Department of Surgery, The Ohio State University Wexner Medical Center, Columbus, OH, United States; ^2^Center for Personalized Diagnostics and Center for Immunotherapy, Vaccines and Virotherapy, Tempe, AZ, United States

**Keywords:** corneal injury, neovascularization, Serp-1, PAI-1, corneal wound repair

## Abstract

**Purpose:** Chemical corneal injuries carry a high morbidity and commonly lead to visual impairment. Here, we investigate the role of Serp-1, a serine protease inhibitor, in corneal wound healing.

**Methods:** An alkaline-induced corneal injury was induced in 14 mice. Following injury, five mice received daily topical saline application while nine mice received Serp-1 100 μL topically combined with a daily subcutaneous injection of 100 ng/gram body weight of Serp-1. Corneal damage was monitored daily through fluorescein staining and imaging. Cross sectional corneal H&E staining were obtained. CD31 was used as marker for neovascularization.

**Results:** Serp-1 facilitates corneal wound healing by reducing fibrosis and neovascularization while mitigating inflammatory cell infiltration with no noticeable harm related to its application.

**Conclusions:** Serp-1 effectively mitigates inflammation, decreases fibrosis, and reduce neovascularization in a murine model of corneal injury without affecting other organs.

**Translational Relavence:** Our study provides preclinical data for topical application of Serp-1 to treat corneal wounds.

## Introduction

Corneal injuries are a commonly cited reason for emergency department visits in the United States (1, 2). Although they are typically non-fatal, they can result in significant morbidity and change in quality of life. Ocular chemical burn in particular, represent an ophthalmic emergency and constitute about 11.5–22.1% of eye injuries (3). The most common causes of ocular chemical burns are household cleaners and building products such as ammonia, sodium hydroxide, and plaster (2). Accidents involving these chemicals require prompt diagnosis and frequently result in hospitalization for treatment (1, 2).

Under normal circumstances, the corneal epithelium acts as a protective barrier to the ocular bulb (2). Intercellular attachments and attachments of epithelial cells to the extracellular matrix through both junctional and non-junctional adhesions, maintain this barrier (4). With damage, epithelial cells undergo apoptosis and are shed in tear films (2). Intercellular junctions are disrupted, and cell-substrate junctions are temporarily replaced with weaker attachments and a provisional extracellular matrix is laid down in preparation for repair (5). Chemical ocular injuries are particularly more deleterious due to the additional oxidative stress they impose on the cornea which not only damages the corneal cells but will also trigger an immune response characterized by inflammation (2, 6). This damage to the cornea causes a keratocyte induced fibrosis which hardens and opacifies the cornea and results in varying degrees of blindness (7). Additionally, while acute inflammation is initially beneficial to the eye, long-term sequalae of inflammation in the cornea include neovascularization which can threaten vision (8).

Serp-1 is a myxoma virus derived SERine Protease INhibitor (SERPIN) with previously demonstrated roles in inflammation, cell migration, wound closure, tissue remodeling and fibrosis (9–12). It is a single-chain glycosylated protein composed of three β-sheets and nine α-helical domains with a strained reactive center loop (RCL) positioned in the carboxy terminus (13). SERPINs are important injury response factors that participate in all stages of injury repair [summarized in our recent review article (14)]. We have previously showed that recombinant Serp-1 protein could be a potential therapeutic agent to reduce aortic balloon angioplasty injury (15), suppress atherosclerotic plaque growth (16), prevent chronic renal (17), and cardiac (18) allograft rejection, promote spinal cord injury repair (19) and full thickness dermal wound healing (10) in rodent models. Here, we investigate the role of Serp-1 in corneal wound healing through immune system modulation.

## Methods

### Animal Corneal Wound Healing Model

Animal husbandry and experimentations were conducted with approval from the Institutional Animal Care and Use Committee (IACUC) at The Ohio State University. All injuries were induced under anesthesia (Henry Schein Isothesia, Isofluorane). Four milliliters of Ibuprofen (Perrigo Basic Care, 100 mg per 5 mL Oral Suspension) was mixed into 400 mL ddH_2_O (Millipore Sigma Milli-Q IQ system) and made accessible to the mice 24 h before the injury and throughout the duration of the entire experiment. A solution of buprenorphine was made by triturating buprenorphine and saline solution (Baxter, 0.9% Sodium Chloride) in a 3:50 ratio by volume. Each mouse received a 100 μL subcutaneous injection of the buprenorphine solution every 12 h following the injury for 72 h.

Fourteen mice (C57BL/6J) were selected for this experiment. A piece of filter paper, 2-mm in diameter, was soaked in 1M NaOH solution and placed on the cornea of the mouse's right eye for 30 s and then rinsed off with 15 mL of saline solution (Baxter, 0.9% Sodium Chloride). The clinical opacity and neovascularization scores were determined using Modified Hackett-McDonald scoring methods for 10 days after the injury (20). After the analysis was complete, the mice were sacrificed, and the eyes were collected for flat-mount and paraffin-embedded staining. The size and depth of the corneal damage was monitored and recorded daily through fluorescein staining and imaging. Ten microliters of fluorescein was placed on the right eye of the mouse and the excess fluorescein was rinsed off with saline. The Kowa SL-17 slit lamp was set to the cobalt blue filter and used to illuminate the eye and excite the fluorescein particles so that they could be captured by a point and shoot camera (Samsung ST150F). Images were obtained once per day.

For treatment of the corneal wounds, the mice were divided into two experimental treatment groups. One group received a control treatment of 10 μL of phosphate-buffered saline (PBS) applied topically for 10 min. The second group not only received a 10 μL (0.1 μg/μl) topical treatment of recombinant Serp-1 protein but also a subcutaneous injection of recombinant Serp-1 protein (0.1 μg/μl) in a dosage equivalent to 100 ng per gram of body weight (recorded every morning) based on our previous publication (10). Treatments were applied twice daily for a total of 10 days following the injury. Mice body weights and eating habits were monitored daily and used as indicators of health status. Heart, lung, kidney, liver, splenic tissues were collected at the completion of the experiment and evaluated for pathology.

### Histopathology and Immunohistochemistry

The mice were sacrificed following 10 days of observations. The eyes were removed, set in paraformaldehyde (BioWorld, 4% in PBS, pH 7.4) overnight, and then transferred into 70% ethanol. The corneas were dissected from the ocular bulb and placed into a 96-well plate with 100 μL PBS (Thermo Fisher Scientific). Corneas were washed 3 times with 300 μL PBS for 10 min per wash and placed in blocking buffer solution for 2 h at room temperature. Anti-CD31 antibodies (1:100, BD Biosciences Pharmingen) was applied, followed by overnight incubation at 4°C. All corneas were washed 6 times with 1x washing buffer for 1 h at a time at room temperature. Secondary antibodies α-rat 488 (1:500, 1:500, Thermo Fisher Scientific) was applied for CD31(angiogenesis/neovascularization marker. The corneas were then washed 3 times with PBS for 1 h each. Four cuts were made on the cornea to “butterfly” the sample so that the sample could be laid down in a flat manner. Mounting was performed in mounting buffer containing 4′,6′-diamidino-2-phenylindole (DAPI). Additionally, full corneal cross-sections were obtained, embedded in paraffin, and stained using Hematoxylin and Eosin (H&E). Immunofluorescent staining of CD11b [anti-CD11b antibody (Invitrogen, 14-0112-82, 1:200)] was performed as follows: slides were deparaffinized and rehydrated by incubating sequentially in xylene, 100% ethanol, 95, 75, 50% ethanol and PBS. A pressure cooker was used for antigen retrieval. Slides were merged in Tris-EDTA buffer and cooked for 13 min. Primary antibody were applied and incubated at 4°C overnight. Goat anti-rat secondary antibody Alexa-488 (Invitrogen, A11006) were applied and incubated at room temperature for 1 h. All images were captured by a Zeiss LSM 780 confocal microscope and analyzed by ImageJ as described in our previous publication (21).

### Opacity and Neovascularization Scores

A modified Hackett-McDonald scoring was used (20). Image J was used for immunofluorescence image analysis. Vessel density was calculated by tracing the fluorescent, CD31 positive areas in ImageJ then dividing that area by the total cornea area to achieve a percentage for vessel coverage.

### Production of Recombinant Serp-1 Protein

Recombinant Serp-1 protein was produced by Chinese hamster ovary (CHO) cell line protein expression system (Viron Therapeutics Inc., London, ON, CA), as described by our previous publication (10). Sequential column chromatographic separation was employed to purify GMP-compliant recombinant Serp-1 protein. Purity of Serp-1 protein (>95%) was determined by Coomassie-stained SDS-PAGE and reverse-phase HPLC. Endotoxin was confirmed absent from purified Serp-1 by LAL assay (10).

### Statistical Analysis

The data are represented as mean ± standard deviation. Comparisons were made by Student's *t*-test when comparing two experimental groups. The standard deviation of the mean is indicated by error bars for each group of data. A value of *p* < 0.05 was considered significant. All of these data were analyzed using Prism 8 software.

## Results

### Serp-1 Facilitates Corneal Wound Healing by Reducing Fibrosis and Neovascularization

To test the effectiveness of Serp-1 as a treatment for corneal injuries, an alkaline-induced corneal injury was induced in 14 mice. Following injury, a control group of five mice received daily topical saline application while nine mice received Serp-1 (100 ng/ μL concentration, 100 μL topically plus 100 ng/gram body weight subcutaneously) each day. Fluorescein dye and quantitative opacity and neovascularization scores were used to determine the level of fibrosis, neovascularization, and re-epithelialization in the corneas.

**Figure 1:**
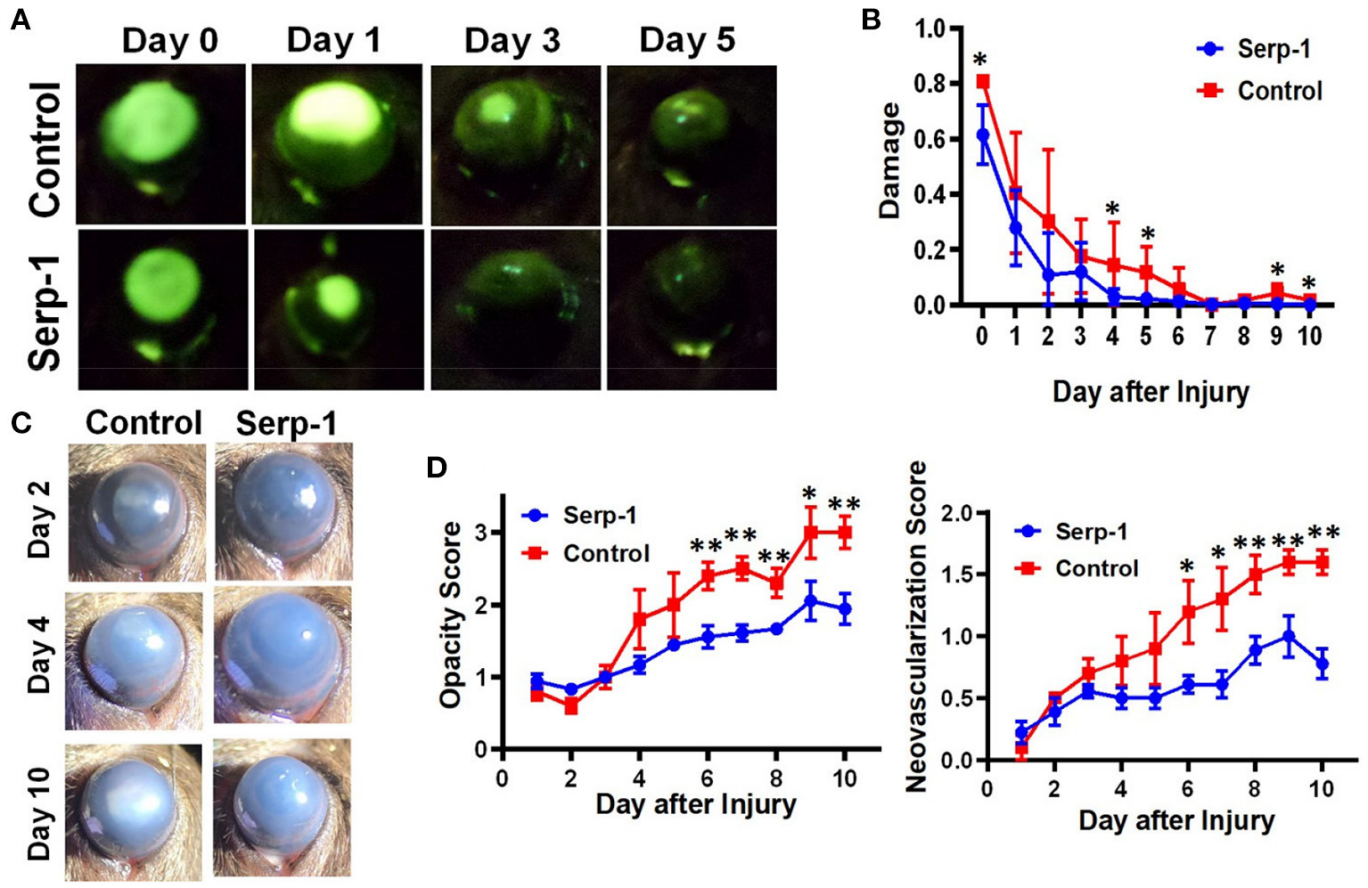
Serp-1 contributes to the corneal wound healing process by limiting the inflammatory response. **(A)** Treatment with Serp-1 shows improved re-epithelialization in mouse corneas with an alkaline induced injury (*n* = 5 for control group and *n* = 9 for Serp-1 group). **(B)** Quantification of fluorescent signal in **(A)** by dividing the fluorescein positive area by total corneal area (data were presented as mean ± S.D. *P < 0.05). **(C)** Bright-field imaging shows reduced fibrosis and encroachment of the cornea by neovascularization in mice treated with Serp-1. **(D)** Quantification of corneal fibrosis and neovascularization using a modified Hackett-McDonald scoring system (data were presented as mean ± S.D. *P < 0.05; **P < 0.01).

**Figure 2:**
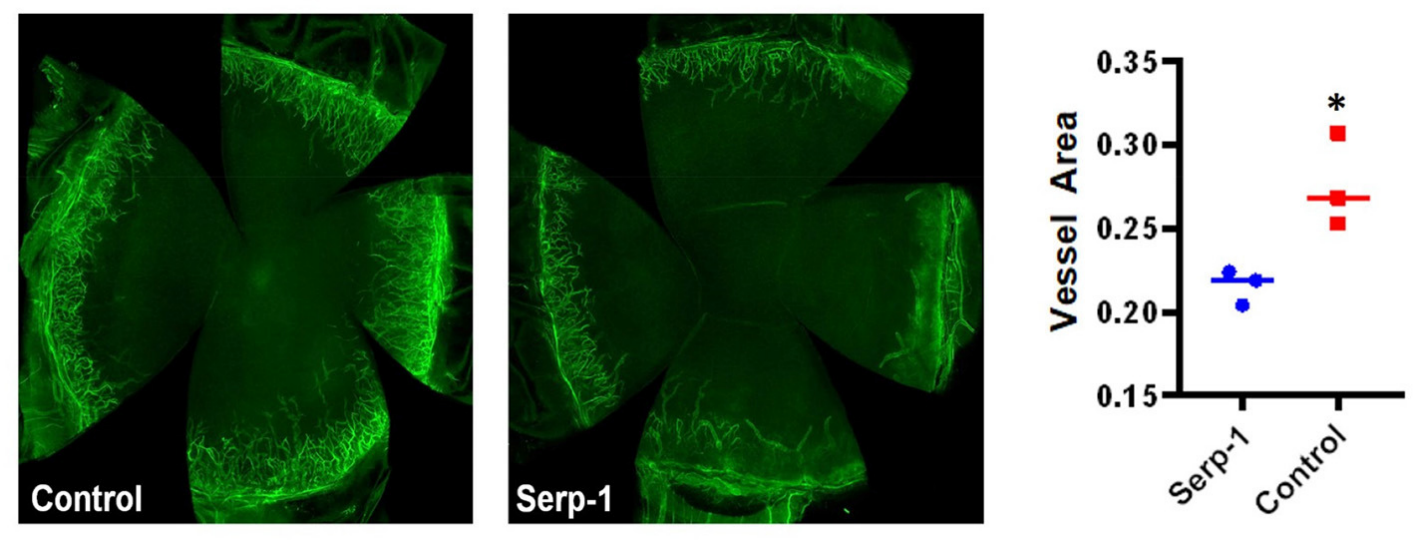
Immunostaining with flat-mounted corneas shows reduced inflammation in Serp-1 treated mice. Immunostaining with flat-mounted corneas shows reduced inflammation in Serp-1 treated mice. Anti-CD31 antibody was applied and samples were mounted in buffer containing 4′,6′-diamidino-2-phenylindole (DAPI). Total vessel area was quantified by dividing the CD31 positive areas by the total cornea area using FijiWin's ImageJ software, with a significant difference reported between the two treatment groups (*n* = 3/group; *p < 0.05).

Fluorescein positive-areas and fluorescence intensity were used as indicators for corneal damage and re-epithelialization. Mice who received Serp-1 treatment exhibited smaller fluorescein-positive areas and lower intensity fluorescence when compared to the mice who received only saline (Figure 1A). A significant difference between the damaged areas was noted by Day 5 following injury (*p* < 0.05) (Figure 1B). Serp-1 treated mice corneas consistently showed reduced opacity and vascularization, with a significant difference in both opacity and neovascularization starting from Day 6 post-injury (Figures 1C,D, ^*^: *P* < 0.05; ^**^: *P* < 0.01). CD31 expression was used as an indicator of the presence of superficial and deep neovascularization in corneal sections. Evaluation of the CD31 staining revealed a reduction in neovascularization in the Serp-1 treated group (Figure 2, ^*^: *P* < 0.05).

### Serp-1 Reduces Rates of Inflammatory Cell Infiltration Into the Cornea

**Figure 3:**
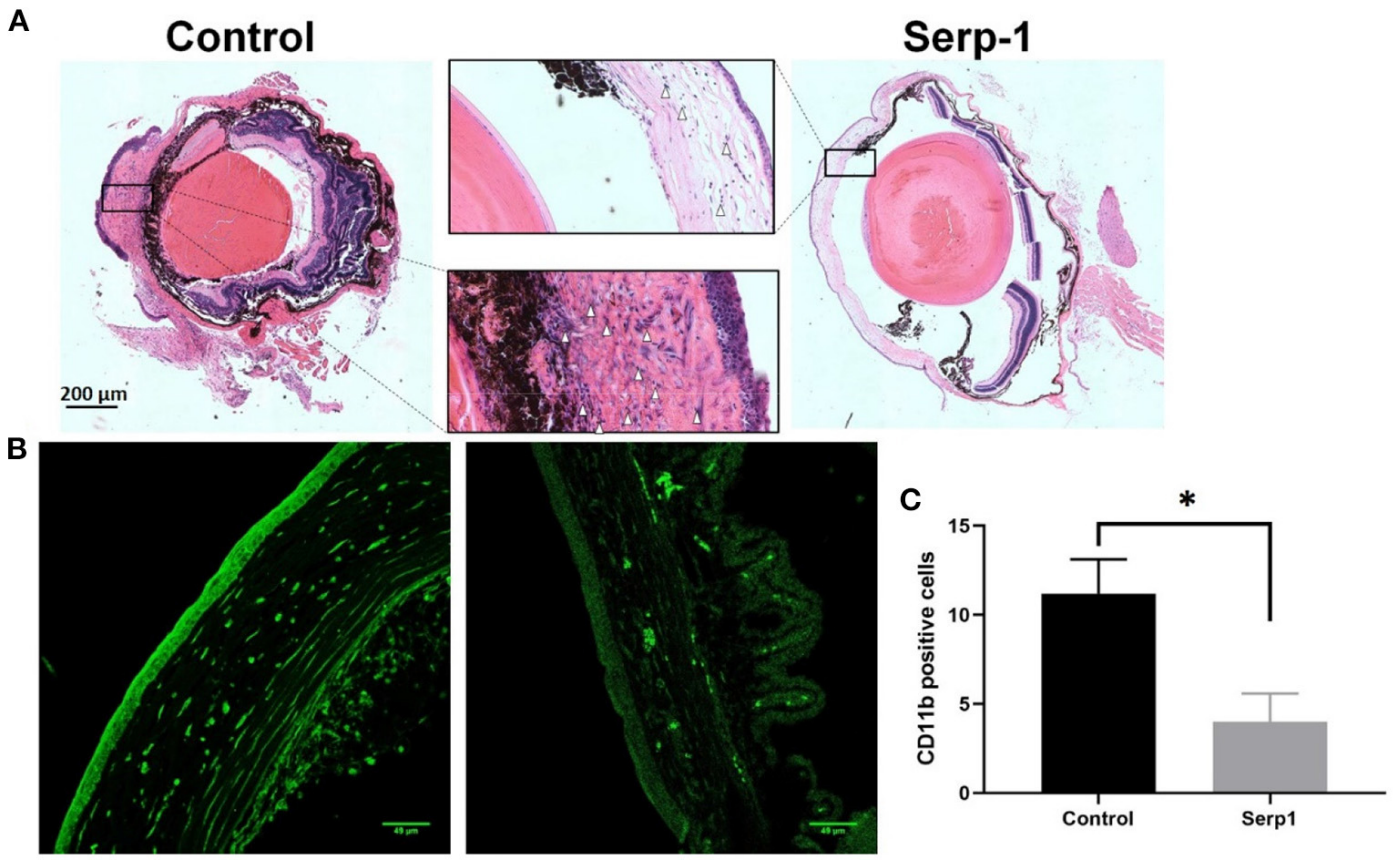
Histochemical analysis of eye cross-sections show reduced rates of inflammatory cell infiltration and swelling of the cornea in Serp-1 treated mice. **(A)** Histochemical analysis of eye cross-sections shows reduced rates of inflammatory cell infiltration and swelling of the cornea in Serp-1 treated mice. Enlarged images of the corneal region of two mice visibly show more swelling and a higher presence of inflammatory cells in the control mice. Inflammatory cell nuclei are stained by the deep-purple spots marked with white triangles in the light pink stromal layer of the cornea. **(B)** To further identify infiltrating immune cells, the slides were stained with CD11b (an immune cell marker, green). **(C)** CD11b positive cells (cells/0.1 mm^2^) were quantified (*n* = 3/group; *p < 0.05).

Histochemical analysis of the enucleated globes of both Serp-1 and control-treated mice was performed following 10 days of observations. Cross-sectional image analysis following H&E staining revealed an obvious reduction of corneal swelling and decreased inflammatory cells infiltration in Serp-1 treated animals (White arrows in Figure 3A). Similarly, CD11b positive immune cells were stained. We found that Serp-1 treatment significantly reduced immune cell infiltration following alkali burn injury (Figures 3B,C, ^*^: *P* < 0.05).

### Repetitive Application of Serp-1 Produces No Obviously Toxic Effects

**Figure 4:**
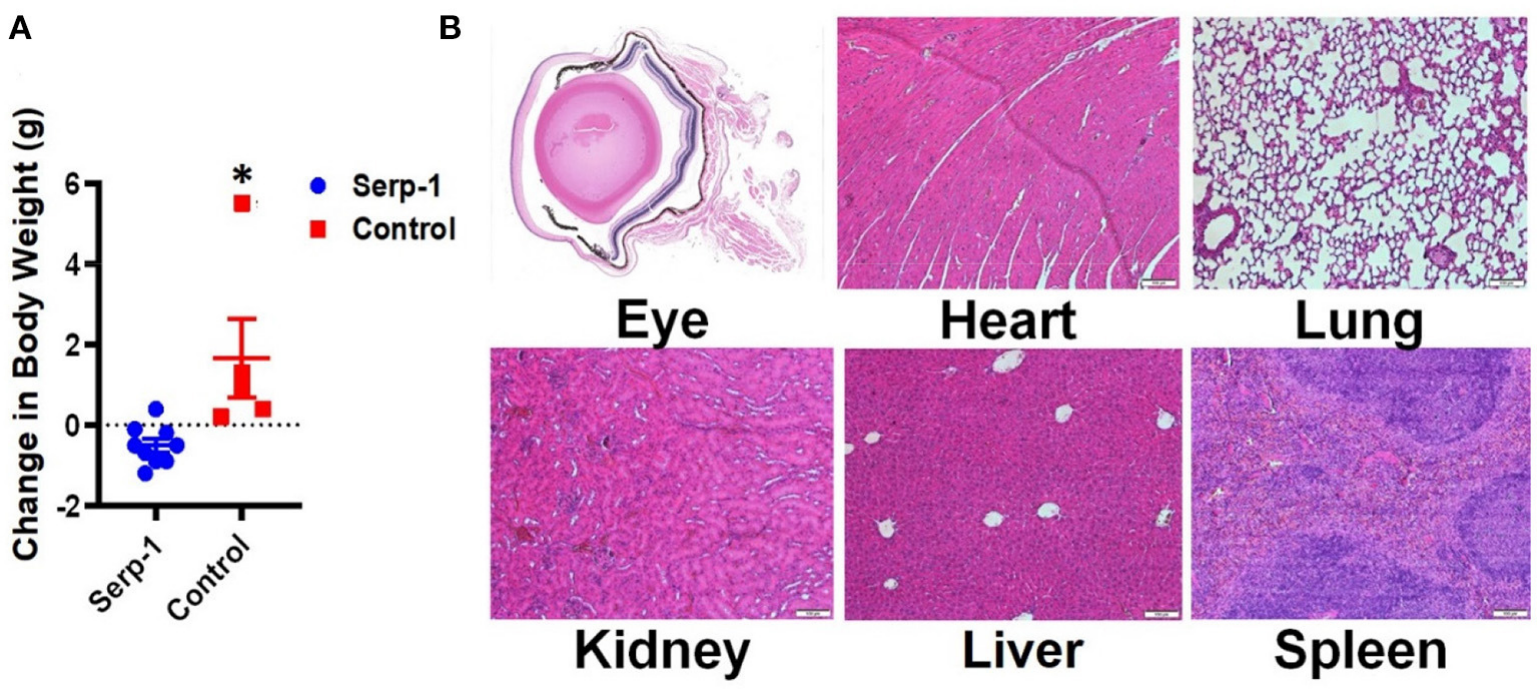
Repetitive application of Serp-1 produces no obviously toxic effects. **(A)** Changes of body weight before and after experiments were measured and calculated (*n* = 5 for control group, *n* = 9 for Serp-1 group. *P < 0.05). **(B)** Histochemical analysis of eye cross-sections shows reduced rates of inflammatory cell infiltration and swelling of the cornea in Serp-1 treated mice. Enlarged images of the corneal region of two mice visibly show more swelling and a higher presence of inflammatory cells in the control mice. Inflammatory cell nuclei are stained by the deep-purple spots marked with white triangles in the light pink stromal layer of the cornea.

Since Serp-1 protein is a virus derived protein, we also checked whether repetitive administration of Serp-1 could induce any adverse effects to the mice. We first checked mouse body weight change after Serp-1 injection. While the mice receiving saline gained body weight, we did observe Serp-1 treatment group had slight reduction of body weight (Figure 4A). However, when we checked major organs of the mice treated with Serp-1, we didn't observe pathologic inflammatory changes as result of the use of Serp-1 (Figure 4B). Thus, further studies will be required to identify the cause of slight body weight loss after repetitive administration of Serp-1 protein in mice.

## Discussions

The application of Serp-1 following corneal alkali induced injury leads to reduced fibrosis and neovascularization, and a decrease in inflammatory cell infiltration. In order to maintain transparency, the cornea must remain avascular and unscarred (22). Since chemically induced corneal injuries rapidly progress to visual impairment, attention has recently turned toward developing treatments for rapid re-epithelialization of the cornea with minimal fibrosis and neovascularization. The novelty of this study is evident in the fact that it demonstrates the therapeutic efficacy of Serp-1 in promoting the natural healing process of the cornea following injury in a mouse model of corneal injury.

We hypothesized that Serp-1 facilitates corneal wound healing by reducing inflammation. An important facet of corneal wound healing is the rate at which the cornea re-epithelialize (22). To monitor re-epithelialization in the mice, fluorescein dye was used daily to stain areas of the mouse cornea where the tight cell-to-cell junctions of the epithelium were compromised. Analysis during a period of 10 days following injury revealed quick healing in mice treated with Serp-1. These results are consistent with the findings from previous work done in evaluating the role of Serp-1 in dermal wound healing (10). The reduced rates of opacity and neovascularization in the Serp-1 treatment group demonstrate a reduced inflammatory response. Additionally, observations from our paraffin-embedded cross sections revealed reduced corneal swelling and improved morphology with Serp-1 treatment following injury which points to an improved rate of corneal epithelialization.

In addition to a fast recovery time, it is essential for the cornea to heal without excessive fibrosis or neovascularization which impede light transmission. Due to its anti-inflammatory properties, it was hypothesized that injured corneas treated with Serp-1 would have reduced amounts of fibrosis and neovascularization. Our data points toward a significant reduction in opacity and neovascularization in animals treated with Serp-1. This was further confirmed through quantitative analysis of the total area of neovascularization in flat-mount staining. Together, these data support the hypothesis that Serp-1 inhibit the inflammatory response in a positive manner that limits fibrosis and scarring that impairs vision.

The immunogenicity of Serp-1 is a legitimate concern due to the fact that it is virally derived. Daily monitoring of mice's body weights and staining of other major organ tissue in these experiments revealed no cytotoxic impact of the repeated application and injection of Serp-1. This finding is entirely consistent with prior research both in pre-clinical or clinical studies wherein Serp-1 demonstrated no significant toxicity and no neutralizing antibodies were detected in the Phase 2 clinical trial in patients with coronary stent implant (23). Future directions as a result of this analysis should include evaluation in larger samples, evaluation of topical applications alone, analysis of interactions with the uPA, MMP proteases after corneal injury and serp-1 treatment, evaluation in other primates, and consideration for clinical trials in humans for an improved understanding of the safety and efficacy of the protein. Further studies should also aim to investigate the molecular mechanism of action of Serp-1 in its anti-inflammatory role. Finally, a recent publication from our group demonstrated that administration of recombinant M-T7 protein (another Myxoma virus protein) could also accelerate dermal wound healing (24). This provides an additional example of a Myxoma virus derived protein in facilitating tissue repair. Thus, future investigations are needed to explore potential Myxoma virus proteins with tissue repair properties.

There is a similar study by Liu et al. elegantly showed topical application of serpinA3K could promote alkali induced corneal wound healing via inhibiting neovascularization and inflammation (25). Thus, it would be interesting to compare the beneficial effects between mammalian serpin and viral serpin in corneal wound healing.

While our study showed beneficial effects of administration of Serp-1 to treat corneal wound in mice, there are some limitations that we will address in our future studies. First, our control treatment group did not use an inactive mutant protein, thus, the beneficial effect we observed in Serp-1 group might be simply due to general protein effect. Our previous publication identified an inactive Serp-1 mutant (26), which would be a preferred negative control for our future study. We are currently working on development of purification protocol for the GMP level mutant Serp-1 for our *in vivo* animal studies in the future. Second, one of direction of our future studies will focus on dissecting the weight loss observe in Serp-1 treatment group. For example, different doses of Serp-1 should be tested and different administration methods should be compared (topical application, intravenous injection, intraperitoneal injection and subcutaneous injection, etc.). Finally, comprehensive toxicology analysis should be included, such as inflammatory cytokine analysis, liver function analysis, potential cardiovascular function analysis and systemic/metabolic disruption of adipose tissue functions or circulating lipids level should be measured.

## Conclusions

Serp-1 can modulates and enhance the corneal wound healing response. The protein can effectively mitigate inflammation, decrease fibrosis, and reduce neovascularization in the cornea following alkaline-induced injuries in a murine model. Further studies to investigate the biologic mechanism of action of Serp-1 are needed to further outline its role, its safety profile, and define its potential as an alternative to standard of care in the treatment of corneal injuries.

## Data Availability Statement

The raw data supporting the conclusions of this article will be made available by the authors, without undue reservation.

## Ethics Statement

The animal study was reviewed and approved by IACUC of the Ohio State University.

## Author Contributions

BJ, OG, MS, BG, SW, and HZ performed the experiments. ARL and HZ designed the studies. BJ, OG, and HZ analyzed the data. BJ, OG, DB-M, JRY, ARL, and HZ wrote the paper. All authors contributed to the article and approved the submitted version.

## Conflict of Interest

The authors declare that the research was conducted in the absence of any commercial or financial relationships that could be construed as a potential conflict of interest.
